# Phase II clinical trial of sorafenib plus interferon-alpha treatment for patients with metastatic renal cell carcinoma in Japan

**DOI:** 10.1186/s12885-015-1675-1

**Published:** 2015-10-09

**Authors:** Masatoshi Eto, Yoshiaki Kawano, Yoshihiko Hirao, Koji Mita, Yoichi Arai, Taiji Tsukamoto, Katsuyoshi Hashine, Akio Matsubara, Tomoaki Fujioka, Go Kimura, Nobuo Shinohara, Katsunori Tatsugami, Shiro Hinotsu, Seiji Naito, Japan RCC Trialist Collaborative Group (JRTCG) investigators

**Affiliations:** 1 Department of Urology, Faculty of Life Sciences, Kumamoto University, 1-1-1 Honjo, Chuo-ku, Kumamoto, 860-8556 Japan; 2 Department of Urology, Nara Medical University, 840 Shijo-cho, Kashihara, Nara 634-8521 Japan; 3 Department of Urology, Hiroshima City Asa Hospital, 2-1-1 Kabeminami, Asa, Kita-ku, Hiroshima, 731-0293 Japan; 4 Department of Urology, Tohoku University Graduate School of Medicine, 2-1 Seiryo-machi, Aoba-ku, Sendai, 980-8575 Japan; 5 Department of Urology, Sapporo Medical University, S1 W17, Chuo-ku, Sapporo, 060-8556 Japan; 6 Department of Urology, National Hospital Organization Shikoku Cancer Center, 160 Kou, Minamiumemoto-chou, Matsuyama, 791-0280 Japan; 7 Department of Urology, Institute of Biomedical & Health Sciences, Hiroshima University, 1-2-3 Kasumi, Minami-ku, Hiroshima, 734-8551 Japan; 8 Department of Urology, Iwate Medical University School of Medicine, 19-1 Uchimaru, Morioka, 020-8505 Japan; 9 Department of Urology, Nippon Medical School, 1-1-5 Sendagi, Bunkyo-ku, Tokyo, 113-8602 Japan; 10 Department of Urology, Hokkaido University Graduate School of Medicine, Kita 15, Nishi 7, Kita-ku, Sapporo, 060-8638 Japan; 11 Department of Urology, Graduate School of Medical Sciences, Kyushu University, 3-1-1 Maidashi, Higashi-ku, Fukuoka, 812-8582 Japan; 12 Department of Phamacoepidemiology, Graduate School of Medicine and Public Health, Kyoto University, Yoshida-Konoe-sho, Sakyo-ku, Kyoto, 606-8501 Japan

**Keywords:** Sorafenib, Interferon-alpha, Renal cell carcinoma

## Abstract

**Background:**

To improve antitumor effects against metastatic renal cell carcinoma (mRCC), use of molecular target-based drugs in sequential or combination therapy has been advocated. In combination therapy, interferon (IFN)-α amplified the effect of sorafenib in our murine model (J Urol 184:2549, 2010), and cytokine-treated mRCC patients in Japan had good prognoses (Eur Urol 57:317, 2010). We thus conducted a phase II clinical trial of sorafenib plus IFN-α for untreated mRCC patients in Japan.

**Methods:**

In this multicenter, prospective study, provisionally registered patients with histologically confirmed metastatic clear cell RCC received natural IFN-α (3 dosages of 3 million U per week) for 2 weeks. Only IFN-α-tolerant patients were registered to this trial, and treated additionally with oral sorafenib (400 mg, bid). The primary end point of the study was rate of response (CR + PR) to sorafenib plus IFN-α treatment assessed using RECIST v1.0. The secondary end points were disease control rate (CR + PR + SD), progression free survival (PFS), overall survival (OS), and safety of the combined treatment. PFS and OS curves were plotted using the Kaplan-Meier method.

**Results:**

From July 2009 to July 2012, a total of 53 untreated patients were provisionally registered, and 51 patients were finally registered. Rate of Response to the combined therapy of sorafenib plus IFN-α was 26.2 % (11/42) (CR 1, PR 10). The median PFS was 10.1 months (95 % CI, 6.4 to 18.5 months), and the median OS has not been reached yet. The combined therapy increased neither the incidence of adverse effects (AE) nor the incidence of unexpected AE. A limitation was that a relatively high number of patients (9 patients) were excluded for eligibility criteria violations.

**Conclusion:**

Our data have demonstrated that sorafenib plus IFN-α treatment is safe and effective for untreated mRCC patients.

**Trial registration:**

UMIN000002466, 9^th^ September, 2009

**Electronic supplementary material:**

The online version of this article (doi:10.1186/s12885-015-1675-1) contains supplementary material, which is available to authorized users.

## Background

Much attention has been paid to molecular target-based drugs including vascular endothelial growth factor (VEGF) tyrosine kinase inhibitors (TKI) and mTOR inhibitors (mTORi) [1–3], all of which were approved by the United States Food and Drug Administration (USFDA) for treatment of advanced renal cell carcinoma (RCC). Although these agents drastically improved progression free survival (PFS) or overall survival (OS) in patients with metastatic RCC (mRCC) compared with immunotherapy or placebo controlled therapy [1–3], they also had limitations, including the rarity of a complete response (CR) [4], rapid progression soon after drug cessation (so-called “rebound phenomenon”) [5], the development of resistance [6], etc. To improve the antitumor effects of molecular target-based drugs, their sequential or combined use has been advocated. In daily clinical practice, we have been using sequential treatments after the failure of previous target-based drugs. On the other hand, the rationale for combination therapy is inhibition of either a single pathway (vertical blockade) or different pathways (horizontal blockade) in order to increase efficacy and reduce toxicity [7].

In combination therapy, although combining 2 targeted agents failed to induce clinical activity due to high incicdence of toxity [8], the additive effect of interferon-alpha (IFN-α) on sorafenib has been recently reported in phase I and II clinical studies of mRCC patients [9–11], and in our study using a murine model [12]. A more recent study demonstrated the good efficacy and tolerability of sorafenib plus frequent low-doses, but not standard doses, of IFN-α [13]. Furthermore, cytokine-treated mRCC patients in Japan have had good prognoses [14]. In addition, the prognosis of mRCC patients in Japan who were initially treated with IFN-α and then with molecular target-based drugs has also been good [15]. The Japan RCC Trialist Collaborative Group (JRTCG) has thus conducted a phase II clinical trial evaluating sorafenib plus IFN-α in untreated mRCC patients in Japan.

## Methods

### Patients, eligibility criteria, and study design

This study’s protocol was approved by the ethics committees of all the clinical sites (Ethical committee for clinical research/medical technology of Faculty of Life Sciences Kumamoto University, Contracted research review committee of Hokkaido Cancer Center, Ethical review committee for clinical research of Kagoshima University Medical and Dental Hospital, Ethical review committee Hirosaki University Graduate School of Medicine and School of Medicine, Institutional Review Board of Asahikawa Medical University, Ethical review committee of Sapporo Medical University, Ethical review committee of Shikoku Cancer Center, Ethical committee of Hamamatsu Medical University, Institutional Review Board of Nippon Medical University, Ethical committee of Isezaki Municipal Hospital, Ethical committee of Sunagawa City Medical Center, Ethical review committee B of Jichi Medical University, Ethical review committee of National Defense Medical College, Ethical committee of Harasanshin Hospital, Ethical review committee of National University Corporation Osaka University Hospital, Contracted clinical research review committee of Nagoya University Graduated School of Medicine, Ethical committee of Hiroshima City Asa Hospital, Ethical committee of Yokohama City University Hospital, Medical Ethical committee of Kobe University Graduated School of Medicine, Clinical trial ethical review committee of Kyusyu University Graduated School of Medicine, Medical Ethical committee of Kyoto University Graduated School of Medicine, Clinical trial ethical review committee of Tsukuba University Hospital, Clinical trial ethical review committee of Hiroshima University, Investigator sponsored clinical research review committee of Hokkaido University Graduated School of Medicine, Ethical committee of Tokyo Women's Medical University, Ethical committee of Tohoku University Graduated School of Medicine, Medical Ethical committee/Clinical Research Review Committee of Kurashiki Central Hospital, Medical department Ethical committee of Keio University, Clinical Research Review Committee of Nara Medical University, Bioethics committee of Dokkyo Medical University, Clinical research ethical review committee of Tokushima University Hospital, North Kyusyu cooperative institutional review board committee, Research Ethics Committee of Miyazaki University, Institutional review board committee of Tenri Hospital, Ethical committee of Iwate Medical University, Ethical committee of Miyazaki Prefectural Miyazaki Hospital, Ethical review committee of Nigata Cancer Center). All patients gave written informed consent. The eligibility criteria include: age ≥20 years old; histologically-confirmed metastatic clear cell RCC with nephrectomy; at least 1 measurable lesion on CT as defined by Response Evaluation Criteria in Solid Tumors (RECIST) v.1.0 [16]; performance status of 0–1 according to the Eastern Cooperative Oncology Group (ECOG) guidelines; life expectancy of at least 12 weeks; no previous history of chemotherapy, cytokine therapy, or molecularly targeted drug therapy (but patients who used IFN-α <6 months as post-nephrectomy adjuvant therapy for primary tumors were eligible), and adequate functions of major organs.

In this multicenter, prospective study, provisionally registered eligible patients received natural IFN-α (3 dosages of 3 million U per week for 2 weeks). Only patients who could tolerate IFN-α treatment were registered to this trial, and oral administration of sorafenib (400 mg bid) was added to IFN-α treatment. IFN-α was interrupted if patients developed IFN-α side effects including grade 3 or higher influenza-like symptoms, depression, decreased leukocytes, decreased neutrophils, or decreased platelets. Dose interruption and up to two dose reductions of sorafenib (to 200 mg bid and 200 mg qd) were undertaken for sorafenib-associated grade 3 to 4 hematologic and grade 3 nonhematologic toxicities.

### Study evaluation

The National Cancer Institute Common Terminology Criteria for Adverse Events (CTCAE) v.3.0 was utilized for toxicity assessment. RECIST v.1.0 was used for response assessment [16]. Confirmed partial response (PR) was defined as having two or more documented objective PRs or better a minimum of 4 weeks apart.

The primary end point of the study was the objective response rate (complete response [CR] + PR) of sorafenib plus IFN-α treatment for mRCC patients using RECIST v.1.0. The secondary end points were the disease control rate (CR + PR + stable disease [SD]), progression free survival ([PFS], time from registration to first radiological or clinical progression, or death from any cause), overall survival ([OS], time from registration to death from any cause), and safety of the combined treatment. Response assessments were performed every 8 weeks by the investigators and confirmed by an expert panel. Treatment with sorafenib and IFN-α was continued until progression of disease, symptomatic deterioration, unacceptable toxicity, treatment delay more than 4 weeks for any reasons, or withdrawal of consent.

### Sample size and statistical methods

The primary endpoint was the proportion of patients who achieved an objective response. Point of estimation of objective response and 95 % confidence interval were calculated. The sample size was determined from the results of previous phase II studies [10]. The expected and threshold proportions (30 % and 12 %) were based on the response to sorafenib monotherapy after at least 1 prior cytokine containing therapy in Japan (12.4 %) [17]. With a type I error of 0.05 and type II error of 0.10, a total of 44 evaluable patients were needed. Assuming a loss of 3 patients, the total sample size was estimated to be 50. PFS and OS curves were plotted using the Kaplan-Meier method [18]. All data were collected at Clinical Research Support Center Kyushu (Fukuoka, Japan), and all analyses were based on the data available as of July 31, 2013.

## Results

### Enrollment of patients for the trial and treatment delivery

**Fig. 1:**
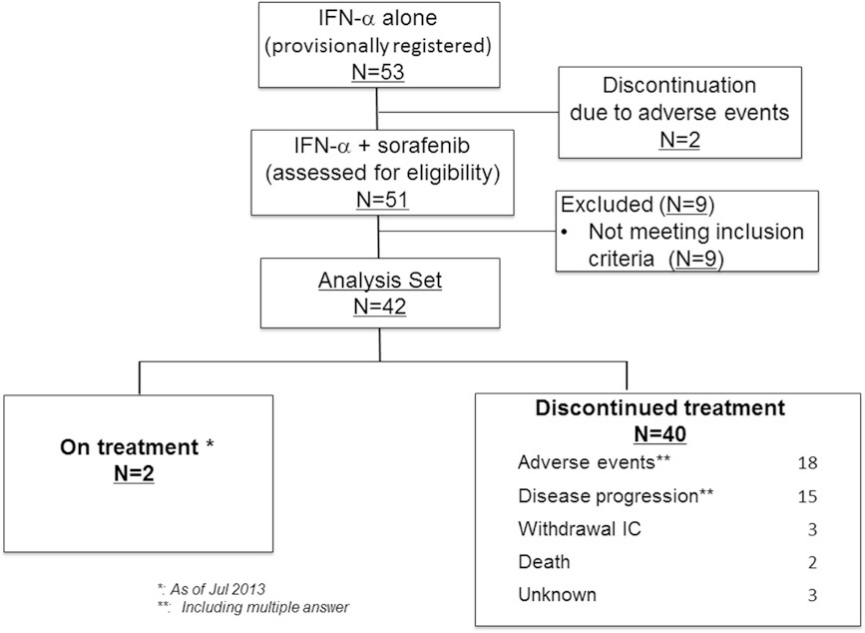
The flowchart of phase II clinical trial of sorafenib plus IFN-α for untreated mRCC patients in Japan

From July 2009 to July 2012, a total of 53 untreated patients were provisionally registered, and 51 patients were finally registered to this trial of sorafenib plus IFN-α (Fig. 1) according to the estimated sample size. Nine patients were excluded because of violation of eligibility criteria, and 42 patients were judged to be eligible. The clinical characteristics of these patients are shown in Table 1. Thirty-five patients were male. The median age of all patients was 64.5 years old (range 37–78). The PS in 37 and 5 patients was 0 and 1, respectively. Twelve, 28, and 2 patients revealed Favorable, Intermediate, and Poor in MSKCC prognostic risk score, respectively. The tumor in one patient had around 5 % spindle cells. The response rate in the ITT population was assessed using these 42 patients. At last report, 2 patients (4.8 %) remained on the protocol treatment (Fig. 1). Common reasons for treatment discontinuation included adverse events in 42.9 %, disease progression in 35.7 %, and patient’s request in 7.1 %.

**Table 1:** Patient background

		*N*(%)	
Age, year	Median (range)	64.5 (37–78)	
Sex	Male	35	(83.3)
	Female	7	(16.7)
PS	0	37	(88.1)
	1	5	(11.9)
MSKCC prognostic risk	Favorable	12	(28.6)
	Intermediate	28	(66.7)
	Poor	2	(4.8)
Histology	Clear cell	42*	(100)
Nephrectomy	Yes	42	(100)
	No	0	
Metastasis	Lung	32	(76.2)
	Liver	5	(11.9)
	Pancreas	5	(11.9)
	Lymph node	4	(9.5)
	Bone	4	(9.5)

*Including one case with spindle cell components 5 %

### Response

**Fig. 2:**
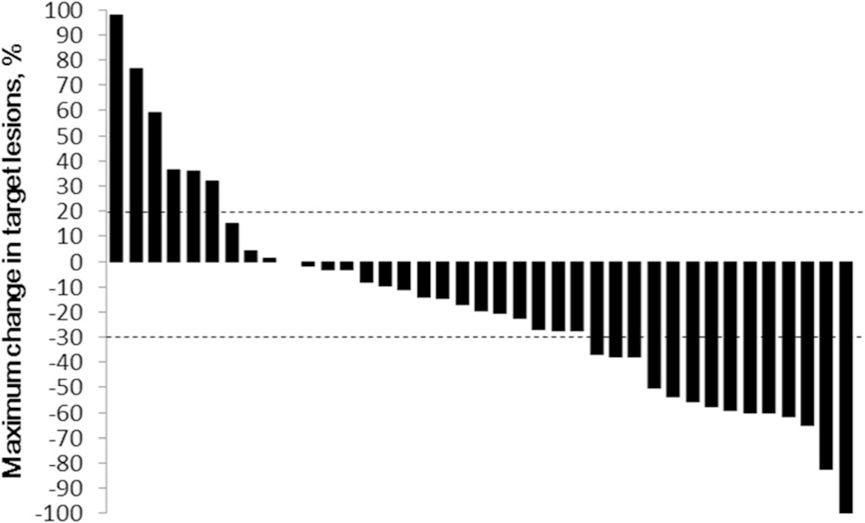
Maximum percentage reduction in target lesions (by Response Evaluation Criteria in Solid Tumors) during treatment with sorafenib plus IFN-α

Response data are shown in Table 2. One complete response and 10 partial responses were observed, for an objective response rate of 26.2 % (95 % CI, 12.9 % to 39.5 %). This study met the primary endpoint, because the lower limit of 95 % CI (12.9 %) exceeded the threshold (12 %). The disease control rate (CR + PR+ SD) was 78.5 % (Fig. 2).

**Table 2:** Response rate

Best response	*N* = 42
CR	1
PR	10
SD	22
PD	6
NE	3
ORR (%)	26.2
DCR (%)	78.5

*CR* complete response, *PR* partial response, *SD* stable disease, *PD* progressive disease, *NE* not evaluated, *ORR* objective response rate, *DCR* disease control rate

### Progression-free and overall survival analysis

**Fig. 3:**
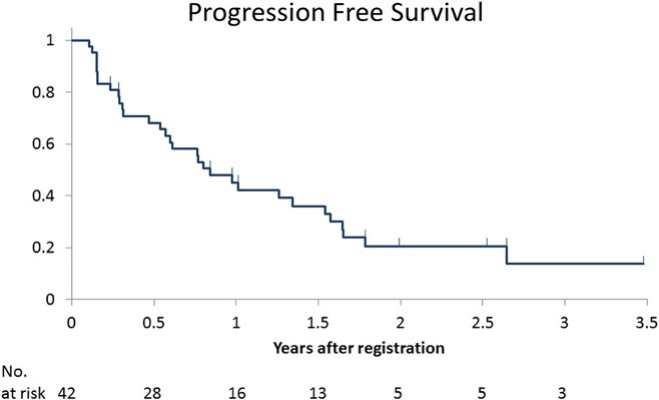
Kaplan-Meier curves of progression free survival (PFS) in mRCC patients treated with sorafenib plus IFN-α. The median PFS was 10.1 months (95 % CI, 6.4 to 18.5)

**Fig. 4:**
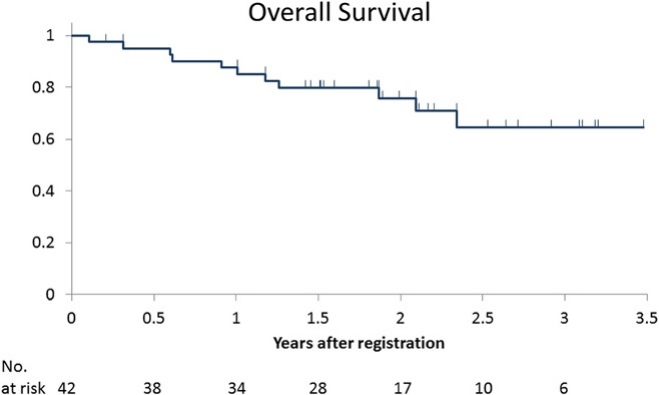
Kaplan-Meier curves of overall survival (OS) in mRCC patients treated with sorafenib plus IFN-α. The median OS has not been reached yet

The median follow-up time of this study was 21.3 months (range, 1.3 to 42.4). The Kaplan-Meier plot of PFS is shown in Fig. 3. The median PFS was 10.1 months (95 % CI, 6.4 to 18.5). The Kaplan-Meier plot of OS is shown in Fig. 4. The OS was good, and the median OS has not been reached yet. Three-year survival rate was 64.5 % (data not shown).

### Toxicity

A summary of common treatment-related adverse events (≥20 %) is shown in Table 3. Common adverse events of each drug were observed in this study. Namely, hand foot skin reaction, rash, lipase elevation, amylase elevation, and hypertension were sorafenib related; malaise, fatigue, thrombocytopenia, leukocytopenia, and pyrexia were IFN-α-related. Depression (related with IFN-α) was observed in 4 patients (9.5 %). Diarrhea, reported in 47.6 % of patients, was a potentially overlapping toxicity of both drugs. No new unexpected adverse event attributable to this combination therapy was encountered.

**Table 3:** Drug associated adverse events (≥20 %)

Adverse events CTCAE ver.3	Any grade		≥ Grade 3	
	IFN-α alone, %	Combined*, %	IFN-α alone, %	Combined*, %
Hand foot skin reaction	0.0	64.3	0.0	21.4
Rash	2.4	52.4	0.0	21.4
Malaise	7.1	57.1	0.0	14.3
Diarrhea	0.0	47.6	0.0	0.0
Thrombocytopenia	2.4	45.2	0.0	7.1
Anorexia	0.0	45.2	0.0	11.9
Leukocytopenia	9.5	47.6	0.0	11.9
Lipase elevation	7.1	31.0	2.4	7.1
Alopecia	0.0	35.7	0.0	0.0
Hypertension	0.0	26.2	0.0	14.3
Amylase elevation	7.1	28.6	2.4	9.5
Fatigue	0.0	26.2	0.0	4.8
Pyrexia	54.8	21.4	0.0	0.0

*Combined therapy of IFN-α + sorafenib

## Discussion

The most important finding of the present study is that sorafenib in combination with IFN-α has been shown to be an effective first-line treatment for mRCC patients in Japan. In accord with the regimen of a recent phase II randomized study [13], our treatment regimen included low-dose (3 million U) IFN-α. Although the response rate (26.2 %) was slightly lower than previous data [13], the median PFS (Fig. 3) was longer in our study (10.1 months) than the previous study [13]. Furthermore, OS was good, and median OS was not reached (Fig. 4). These good results may correlate with the good prognosis of mRCC patients in Japan at the cytokine era [14], or may be ascribed to the better ECOG PS in our study than in the previous study (0–1 vs. 0–2) [13]. Alternatively, the post-treatment after this study may be a potential factor that influenced the good OS, although we have not examined it.

When considering the mechanisms underlying combination therapy with sorafenib plus IFN-α, we need to focus on the role of IFN-α. The main functions of IFN-α are considered to be antiangiogenesis and immune regulation. From the antiangiogenic point of view, the appropriate dose of IFN-α seems to 3 million U, according to Folkman et al. [19]. So far, all [8–10, 20] but one study [21] have demonstrated the effectiveness of IFN-α when combined with molecular targeted drugs (Additional file 1: Table S4). The exception (a randomized phase II study which compared sorafenib alone with a combination of sorafenib and very low dose [0.5 million U twice daily] IFN-α) revealed no advantage for patients in the combination arm, indicating that the selected dose of IFN-α was suboptimal (Additional file 1: Table S4). Taken together, these findings suggest the indispensability of at least 3 million U IFN-α when IFN-α combined with molecular targeted drugs. However, the optimal dose of IFN-α and optimal schedule of administration still need to be determined.

From the immunological point of view, the combination of sorafenib with IFN-α seems to be quite reasonable. Although no immunological assessments were performed in this clinical trial, the underlying immunological mechanisms of this combination therapy of sorafenib plus IFN-α were examined in a murine model in our previous study [12]. Of the 4 groups (untreated, IFN-α alone, sorafenib alone, and sorafenib plus IFN-α) in that study, the sorafenib plus IFN-α group benefited the most [12], and the IFN-α alone and sorafenib plus IFN-α group both had cytotoxic T lymphocyte (CTL) activity and natural killer cell (NK) activity [12]. Interestingly, neither CTL activity nor NK activity was demonstrated in the sorafenib alone group, in spite of the substantial antitumor activity [12]. Taken together, these findings indicate that sorafenib, in the absence of IFN-α, cannot induce the immune response, and thus, IFN-α may have prolonged the CR in the several reported cases (6 %) of the Rapsody study [13] and in the one case (2.4 %) of our study (Additional file 1: Table S4). To overcome the rarity of CR by molecular targeted therapy [4], sorafenib plus IFN-α could be useful, although sorafenib itself is not basically recommended as first line treatment. Furthermore, there are some reports which demonstrate that sorafenib decreases Treg cells in mRCC [22, 23], suggesting that sorafenib could become a candidate drug when combined with immunotherapy in mRCC patients.

We paid much attention to safety issues, with careful monitoring of adverse events. In our study, a high incidence of patients (42.9 %) receiving sorafenib plus IFN-α discontinued treatments due to adverse events (Additional file 1: Table S4). Although the incidence of discontinuation was a little higher than other studies (Additional file 1: Table S4), the dose reduction rate of the combination therapy was almost compatible with other studies (Additional file 1: Table S4). Regarding this point, as we performed this study as an investigator-initiated clinical trial, but not sponsor-initiated clinical trial, more investigators might have treated patients in the style of daily medical practice, resulting in early exchange of molecular targeted drugs. Indeed, a recent post-marketing clinical trial of sorafenib in Japan also demonstrated that a high incidence (40 %) of patients discontinued sorafenib, and started other molecular targeted drugs [24]. However, as shown in Table 3, the common adverse events of each drug but no new unexpected adverse events were observed. Although a previous report suggested a possible decreased incidence of hand foot skin reaction in patients treated with this combination of sorafenib plus IFN-α [10, 11], our study found no such decrease (Table 3). Thus, incidence of adverse events associated with administration of sorafenib plus IFN-α will need to be investigated further.

A major limitation of this study was the relatively small sample size. Furthermore, a relatively high number of patients (9 patients) were excluded for eligibility criteria violations, mostly RECIST criteria violations (data not shown). Since the beginning of the cytokine era in Japan, many urologists have tended to treat patients with even very small metastatic lesions (less than 1 cm), and this may be one reason for the good prognosis of mRCC patients with cytokine therapy in Japan. When we started this prospective trial in 2009, RECIST criteria was not so widely used. As a result, mRCC patients whose largest metastatic lesion was less than 1 cm in diameter were misregistered in this trial. Although we excluded the mRCC patients who did not meet the RECIST criteria, some of them demonstrated objective responses to this sorafenib plus IFN-α regimen (data not shown), implying that the rate of response to this combination therapy would be better than it actually was in this study. The fact that only IFN-α-tolerant patients were registered to this trial could also be a cause of bias. Therefore, a prospective randomized trial comparing sunitinib or pazopanib versus a combination of sorafenib with an optimal dose of IFN-α will be needed to evaluate the efficacy of sorafenib plus IFN-α as first line treatment for mRCC patients.

## Conclusions

In this study, we have conducted a phase II clinical trial of sorafenib plus IFN-α for untreated mRCC patients in Japan. The rate of response to the combined therapy was 26.2 % (11/42) (CR 1, PR 10). The median PFS was 10.1 months (95 % CI, 6.4 to 18.5 months), and the median OS has not yet been reached. Our results have clearly demonstrated that sorafenib plus IFN-α treatment is safe and effective for untreated mRCC patients.

## Additional file

Additional file 1: Table S4. Comparison of the current study with other sorafenib-interferon combination studies and with single-agent sorafenib studies [25]. (XLSX 12 kb)
